# Efficacy of FODMAP Elimination and Subsequent Blinded Placebo-Controlled Provocations in a Randomised Controlled Study in Patients with Ulcerative Colitis in Remission and Symptoms of Irritable Bowel Syndrome: A Feasibility Study

**DOI:** 10.3390/nu14061296

**Published:** 2022-03-18

**Authors:** Dorte Melgaard, Jeanette Sørensen, Johannes Riis, Tine S. Ovesen, Peter Leutscher, Suzette Sørensen, Julie K. Knudsen, Caspar Bundgaard-Nielsen, Jeanette Ejstrup, Ann-Maria Jensen, Mette Borre, Anne L. Krarup

**Affiliations:** 1 Centre for Clinical Research, North Denmark Regional Hospital, 9800 Hjoerring, Denmark; johannes.j@rn.dk (J.R.); p.leutscher@rn.dk (P.L.); suzette.soerensen@rn.dk (S.S.); julie.knudsen@rn.dk (J.K.K.); c.bundgaardnielsen@rn.dk (C.B.-N.); a.maria@rn.dk (A.-M.J.); 2 Department of Clinical Medicine, Aalborg University, 9000 Aalborg, Denmark; apslk@rn.dk; 3 Department of Medicine, North Denmark Regional Hospital, 9800 Hjoerring, Denmark; j.soerensen@rn.dk (J.S.); tine.ovesen@rn.dk (T.S.O.); jeanette.ejstrup@rn.dk (J.E.); 4 Department of Clinical Nutrition, North Denmark Regional Hospital, 9800 Hjoerring, Denmark; 5 Department of Hepatology and Gastroenterology, Aarhus University Hospital, 8210 Aarhus, Denmark; mette.borre@aarhus.rm.dk; 6 Department of Acute Medicine and Trauma Care, Aalborg University Hospital, 9000 Aalborg, Denmark

**Keywords:** ulcerative colitis, inflammatory bowel disease, IBD, irritable bowel syndrome, IBS, low FODMAP diet, FODMAP

## Abstract

Background: Patients with inflammatory bowel disease (IBD) and symptoms of irritable bowel syndrome (IBS) may be intolerant to fermentable carbohydrates (FODMAPs). The aim of this study was to test the feasibility of eliminating and subsequently reintroducing FODMAPs in patients with IBS symptoms as part of the IBD manifestation and to compare the severity of IBS symptoms and pain, bloating and quality of life (QoL). Methods: An eight-week randomised open-label FODMAP elimination with double-blinded, crossover provocations of FODMAP and placebo. Diet patients were on a low-FODMAP diet for eight weeks with blinded two-week provocations after two and six weeks. Questionnaires, blood and stool samples were collected. Results: Patient enrolment was challenging. Nineteen participants were included in the study. Eliminating low FODMAP for two weeks resulted in significant decreases in pain and bloating scores (*p* < 0.003), whereas there were no statistical differences in pain scores between diet patients and controls. Pain and bloating scores increased, returning to baseline levels after two weeks of double-blinded provocations with placebo, (*p* > 0.05). Conclusions: The results document the possibility of performing a randomised controlled study following the gold standard for testing food intolerance with blinding of the Low FODMAP diet. Recruitment of participants was challenging.

## 1. Introduction

One-third of patients with inflammatory bowel disease (IBD) in remission have symptoms of irritable bowel syndrome (IBS) [1,2]. Patients with IBD typically suffer from bloating, abdominal pain, and diarrhoea or constipation, and the term IBS in IBD has been proposed to describe this clinical manifestation of IBD [3,4]. Co-existence of IBS symptoms further deteriorates the quality of life (QoL) in patients with IBD, and effective treatment for relief of the symptoms is still lacking [1,4,5,6,7]. The mechanisms behind IBS in IBD are unknown, but intake of the poorly absorbable fermentable oligo-, di-, and monosaccharides and polyols (FODMAPs) such as fructose, lactose, sorbitol, and mannitol has been found to exacerbate gastrointestinal (GI) symptoms [8]. Furthermore, a diet low in FODMAPs may reduce symptoms in patients with IBS, thus increasing QoL for these patients [9]. 

However, the efficacy of FODMAP alone to reduce symptoms compared to a placebo and nocebo effect has not yet been determined due to the difficulties inherent in diet intervention studies [10]. Previous studies have shown a reduction in symptoms in both patients on placebo and a low-FODMAP diet [4,10]. The challenges related to study design consist of ensuring blinding, creating comparable study logistics compatible with patients’ lives, and prolonging the study period to capture symptom variation over time. Blinding is difficult to achieve in diet intervention studies as patients often guess the blinding status by observing their food and investigating the FODMAP content [11,12,13]. The gold standard for evaluating food intolerance is elimination, provocation, elimination, and provocation in a double-blinded and placebo controlled set-up [14]. The efficacy of FODMAPs has not yet been tested in such a design in patients with IBS in IBD.

The aim of the present study was to test the feasibility of eliminating and subsequently reintroducing FODMAPs in patients with *IBS in IBD*. Moreover, to compare the severity of IBS symptoms and subsequent pain, bloating and QoL in patients receiving either a FODMAP diet or placebo.

## 2. Materials and Methods

This study was an eight-week randomised open-label FODMAP elimination trial with double-blinded, crossover provocations of FODMAP or placebo, compared to a control group. The efficacy of low FODMAP elimination and provocation on IBS symptoms was investigated in patients with ulcerative colitis in deep remission with concurrent IBD symptoms. The study complied with the declaration of Helsinki and was conducted at the North Denmark Regional Hospital, Hjoerring, and Aalborg University Hospital in Denmark. The local data authorities and ethics committee approved the database and study protocol (N-20180005), and the study was registered at ClinicalTrials.gov (accessed on 30 January 2022) under the identifier NCT02469220. Oral and written informed consent was obtained from all participants.

### 2.1. Participants

Patients were recruited from the gastroenterology outpatient clinics at North Denmark Regional Hospital, and Aalborg University Hospital with a combined uptake area of 590,439 people. Inclusion started in July 2018 and was ended in August 2020. The inclusion criteria were age between 18 and 70 years, Ulcerative Colitis (UC) in remission, fulfilment of the ROME IV criteria for the diagnosis of co-morbid IBS, no or stable medical treatment with 5-aminosalicylic acid or biological therapy. Exclusion criteria were intake of a low-FODMAP diet within six weeks before study inclusion; atypical UC with right-sided inflammation and calprotectin >50 unless a normal colonoscopy documented remission; Clostridium difficile infection; lactose intolerance; comorbid coeliac disease or elevated transglutaminase; pregnancy; antibiotic treatment up to six weeks before inclusion; other treatment for UC than stated above; flare in UC; eating disorder; unable to follow the low-FODMAP diet for any reason; other disease than IBD explaining the IBS symptoms; medication explaining the symptoms. Patients fulfilling the above-mentioned inclusion criteria underwent sigmoidoscopy or colonoscopy if this had not been performed within the last three months. After screening, written consent was confirmed.

A sample size calculation using data from a previous study in patients with IBD was performed [15]. According to this calculation a minimum difference in the symptom score on a VAS of 2.5, a standard deviation of 2.3 with 80% power and α = 0.05 a total of 45 patients, 15 in each group, were needed.

### 2.2. Measurements

**Figure 1:**
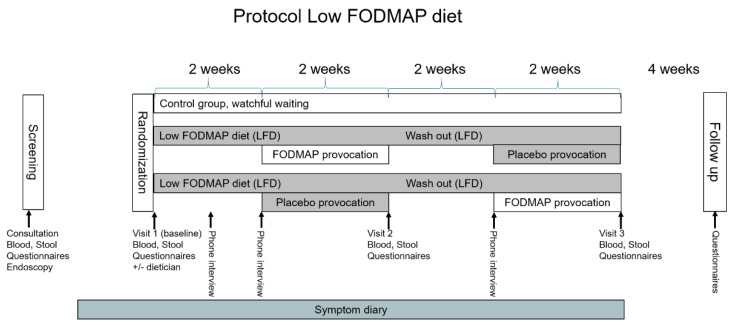
Figure 1 shows the protocol. After randomisation, 1/3 was a watchful waiting control group, and 2/3 of patients were placed on an open label low-FODMAP diet and provoked double-blinded with placebo and FODMAPs, respectively in a cross-over fashion. Between provocations there was a two-week low-FODMAP diet wash out. During the study, patients had three identical consultations with questionnaires, blood and stool sampling. A symptom diary was filled in daily throughout the entire study period.

The timeline of the study is illustrated in Figure 1. Included patients were randomised to the study at a 1:1:1 ratio. The randomisation code was computer generated and kept in a locked room, only accessible to the kitchen supervisor. The food supplements were delivered in a box containing information only on the randomisation number and time period.

As illustrated in Figure 1, two groups received the low-FODMAP diet. The provocation food supplements contained either FODMAP levels typical of a Danish diet or placebo. The control group (Figure 1) underwent the same visits, phone calls, questionnaires, tests, blood tests and faecal samples, but no dietary intervention, as in the intervention group. After study completion, the control group was offered instruction in the diet if interested. All patients had three visits in the outpatient clinics after the randomisation (Figure 1). An overview of tests and questionnaires completed in connection with the visits can be seen in Table 1. In the week before visit 1, Biopsies for histology was sampled from rectum and sigmoideum and all screening questionnaires were filled in. During visit 1, randomisation of the patients was performed, and all patients completed the remaining questionnaires electronically. Those randomised to dietary intervention were instructed by a certified dietician on how to adhere to the low FODMAP diet, and frozen food supplements were handed out for the first period. The lists of foods to be included or excluded were reviewed with the patients. One week after visit 1 and visit 2, respectively, an investigator contacted each participant by telephone to clarify any questions. The faecal samples for visits 1–3 were collected and frozen to −18° at home and carried in a thermo bag with frost elements to the hospital.

Data were collected and managed using Research Electronic Data Capture (REDCap) tools hosted by North Denmark Region. REDCap is a secure, web-based application designed to support data capture for research purposes [16,17].

### 2.3. Food Provocations

The recipes for the provocation foods complied with the low-FODMAP principles before addition of either FODMAPs or placebo [9]. Sucrose was used as placebo as FODMAPs have a sweet taste. Blinded taste testing of the finalised provocation foods was performed by the study personnel, dietitians and kitchen staff to ensure identical smell, looks, taste and consistency. The provocation foods were added to the diets of patients, respectively: A 2 dL breakfast smoothie, 100 g low dark rye bread for lunch, and 250 g soup for dinner. Calculations of daily FODMAP intake were performed using the FODMAP calculator from Monash University [18]. The amount of FODMAPs in a normal Danish diet was estimated among 20 randomly selected, healthy Danish volunteers, registering their food intake in detail over one week (data not shown). The amount was 30 g of FODMAPS/day. For the provocation foods, 5 g was subtracted, as this is the intake on a low-FODMAP diet. The amount of FODMAPs in the provocation foods were 25 g in total (fructose: 3.23 g, sorbitol: 2.28 g, mannitol: 0.40 g, lactose: 14.42 g, fructans: 3.58 g, galacto oligosaccharide (GOS): 0.85 g). 

### 2.4. Questionnaires

The following questionnaires were filled in by the participants to assess disease severity: Mayo Score [19], Rome IV Criteria for IBS [20], Irritable Bowel Syndrome Severity Scoring System (IBS-SSS) [21,22,23,24], IBS-specific Gastrointestinal Symptom Rating Scale (GSRS-IBS) [25], Irritable Bowel Syndrome Adequate Relief (IBS-AR) [26,27,28] and Patient Health Questionnaire (PHQ-15)—Somatisation [29]. Finally, each participant filled in a symptom diary, starting one week before randomisation, and running throughout the study for a total of 56 days. The diary consisted of a 100 mm long visual analogue scale (VAS), to score average daily pain, maximum pain and bloating, as well as stool frequency and consistency. Two questionnaires were used to identify anxiety and depression: The Hospital Anxiety and Depression Scale (HADS) [22] and the Visceral Sensitivity Index (VSI)-GI specific anxiety [23,24]. Health-related quality of life was measured with Short Form 36 Health Survey (SF-36) [30].

Diet registration and FODMAP food frequency registration were performed three days before each outpatient visit. Food item and amount (weight, volume) was registered for two weekdays and one day in a weekend. The questionnaire consisted of five pages of specified foods and drinks containing FODMAPs. The amount of specific FODMAPs was subsequently calculated using the FODMAP calculator [18].

Patients were asked which provocations they thought they had received during the past two weeks and if they had experienced that the provocations decreased, increased or did not change their pain. Compliance with the provocation foods was checked by counting of the remaining food supplements after provocation.

Laboratory analyses of blood samples at each of the three visits were analysed for C-reactive protein, white blood cell count, total iron, transferrin, ferritin, haemoglobin (whole blood), folate, cobalamin and red blood cell volume. Faecal calprotectin was extracted using BÜHLMANN CALEX caps (Bühlmann Laboratories AG, Schönenbuch/Basel, Switzerland) and measured using the BÜLMANN fCAL turbo method. Gut microbiota analysis was performed on faecal samples as described previously [31]. Briefly, bacterial DNA was extracted using a QIAamp PowerFecal Pro DNA kit (QIAGEN, Copenhagen, Denmark) according to manufacturer’s instructions. The resulting DNA was investigated on the Illumina MiSeq platform (Illumina, San Diego, CA, USA) by 16S rRNA gene sequencing targeting the hypervariable V4 region.

### 2.5. Outcomes

The primary outcome was the numerical change in IBS-SSS score after four and eight weeks of diet and either provocation with low FODMAP (placebo provocation) or FODMAPS (FODMAP provocation), respectively. Secondary outcomes were (1) changes in pain and bloating scores from daily symptom diaries and (2) changes in QoL.

### 2.6. Statistical Analysis and Bioinformatics

We used numbers and percentages to present categorical variables. Continuous variables were reported as medians and interquartile ranges. Comparing the sum of the primary outcome in the two periods between groups showed no significant carry-over effects. Differences in outcomes were tested by paired Wilcoxon’s signed-rank tests and Chi-squared tests as appropriate. Symptom diaries were averaged across each week, and a graphical representation of the mean difference between each week and the baseline score was presented. 

All analyses were performed as complete-case intention to treat analyses. A *p*-value below 0.05 was considered statistically significant. For microbiota data, bioinformatics was performed using an Usearch11 pipeline as previously described [31]. For microbiota data, alpha diversity was compared using repeated-measures ANOVA, while beta diversity was investigated using principal coordinate analysis (PCoA) on the Bray–Curtis dissimilarity. All remaining statistical analyses were performed using R version 3.5.3 [32].

## 3. Results

**Figure 2:**
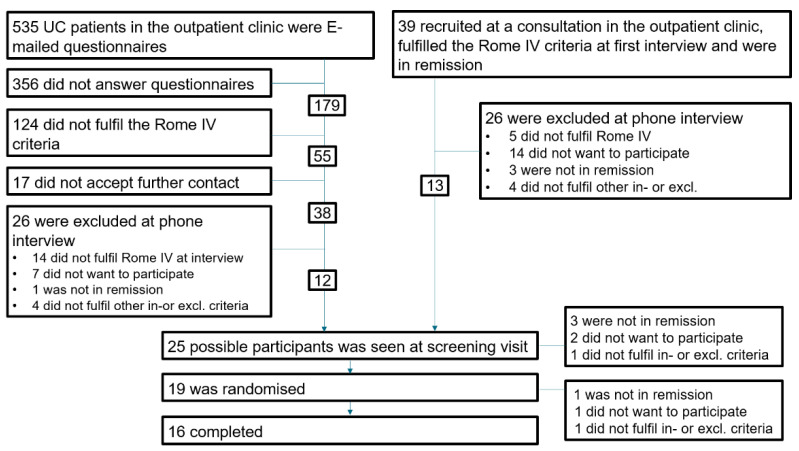
This figure shows the inclusion process. Half of the patients were found via questionnaires sent out electronically, and the other half were recruited at planned outpatient consultations. The questionnaires were not filled in by 67% of patients. Of those who completed the questionnaires, 31% fulfilled the Rome IV criteria for IBS; however, of those accepting contact, 37% did not fulfil the Rome IV criteria at the time of the interview.

The Figure 2 shows the inclusion of patients. A total of 34% of the patients invited from the outpatient clinics completed the questionnaires. Although 31% of the patients from the outpatient clinics complied with the Rome IV criteria in the questionnaire, one-third of them did not meet the criteria at the interview due to, e.g., a flare of UC or misunderstanding of the questionnaire. Patient’s demographics are shown in Table 2 (combined) and Supplementary Table S1.

### 3.1. Feasibility of Blinding and Adherence to Low FODMAP Diet

All patients on a low-FODMAP diet self-reported adherence to the low-FODMAP diet and intake of the food supplement treatment (Table 3). There was a significantly decreased intake of FODMAPs in the diet groups, but not the control group (Table 3). Patients did not guess blinding status (Table 3). 

### 3.2. Primary Endpoint

**Figure 3:**
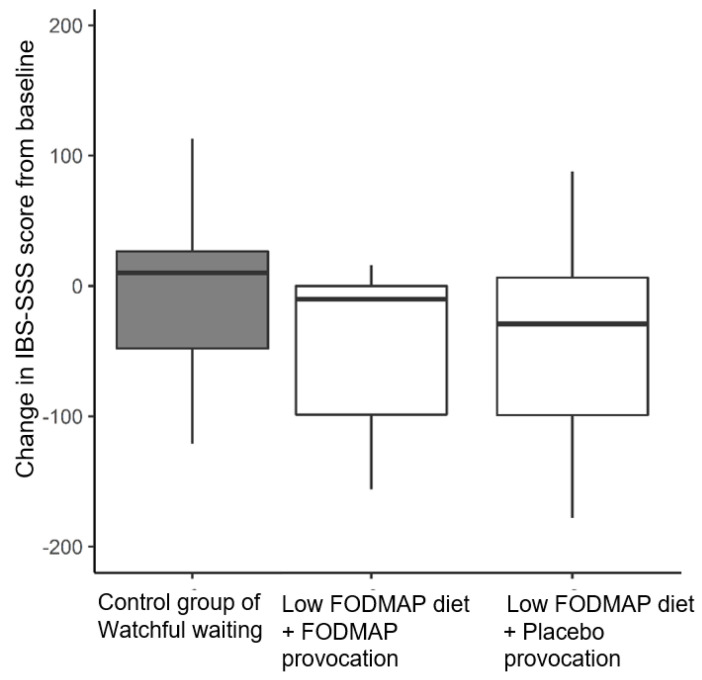
While on watchful waiting, the controls did not change their IBS-SSS scores from baseline. Patients on the low-FODMAP diet experienced no reductions in their IBS-SSS score after the combined time period of first open-label low-FODMAP diet and subsequent provocations with either placebo or FODMAPS. The lack of difference was a consequence of the provocation effects cancelling the initial dietary effect.

There was no change in IBS-SSS scores after low FODMAP diet and placebo provocation combined (*p* > 0.99, Figure 3). Patients in the control group did not report change in their IBS-SSS scores from baseline either. Hence, IBS-SSS scores in all three groups (diet, diet (placebo) and control group) were comparable (Figure 3).

### 3.3. Secondary Endpoints

**Figure 4:**
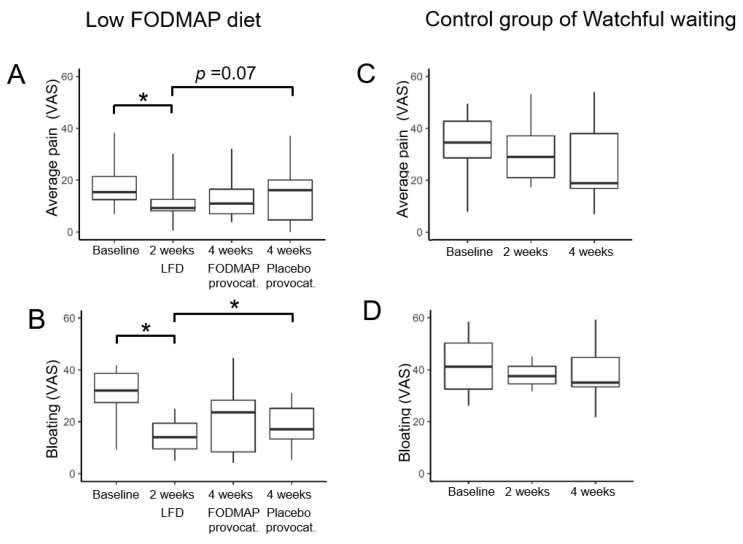
The results of the symptom diaries showed that watchful waiting in the control group resulted in a 50% pain reduction (**A**) and bloating reduction trend (**B**). In diet groups, two weeks on the open-label low-FODMAP-diet resulted in significant decreases in pain and bloating scores (**C**,**D**). However, after the subsequent two weeks provocations, pain and bloating scores returned to baseline levels regardless of the provocation placebo (*p* < 0.05, nocebo effect) or FODMAPs (**C**,**D**). * *p* < 0.05.

The symptom diary made it possible to distinguish between the effects of diet and provocations on a weekly basis. After two weeks, pain score decreased significantly with 40% (*p* = 0.002) and bloating score with 56% (*p* < 0.001) compared to baseline in the low-FODMAP diet groups (Figure 4A,B). However, when provoked blindly with placebo, symptoms increased and were similar to baseline levels eliminating the initial diet effect (*p* > 0.05, Figure 4A,B). Patients in the control group showed a decreasing trend in the median abdominal pain score of almost 50% (*p* = 0.22, Figure 4C), and in the bloating score by a median of 38% (Figure 4D). These results matched improvements in the diet group while they were on the low-FODMAP diet (*p* = 0.92).

Results for a priori defined secondary outcomes in patients on diet and provocations were similar and no clinically relevant differences were observed. There was no indication of UC flare during the study period as calprotectin levels remained normal for all patients. Blood sample analysis did not show clinically relevant changes. In gut microbiota measures, variations were observed across all groups during the study period, although interpersonal variations superseded the effects of treatment. No treatment-specific effect was observed (Supplementary Figure S1).

## 4. Discussion

This feasibility study is the first randomised study of low-FODMAP elimination with subsequent placebo-controlled, double-blinded cross-over provocation related to symptoms of patients with *IBS in IBD* in deep remission. Recruitment was challenging. The blinding of the provocation foods was effective. This study followed the gold standard of testing for food intolerance. The FODMAP elimination followed by randomised subsequent double-blinded, cross-over reintroduction of FODMAPs was additionally compared to a control group to estimate the effect of participation in the study procedures alone without any intervention. This enabled us to estimate the efficacy of the diet alone with a control group undergoing the same non-diet interventions as the diet groups. The two-week duration of each elimination and provocation of FODMAPs/placebo has, in previous studies, been shown to be an adequate duration for the diet to show efficacy and provocations to provoke symptoms [11]. The two RCTs previously published on the effect of low-FODMAP diet in *IBD-IBS* overlap were of 1 to 14 days in duration, which is problematic as IBS symptoms vary over time. An optimal study would run over months or even years due to symptom fluctuations in IBS. However, the longer the duration of a strict diet and frequent hospital visits, the higher the risk of dropouts, non-compliance, and risk of poor generalisability as patients willing to continue participation could be less and less comparable to the average patient with IBD. The choice of two weeks of diet or provocation was a compromise to optimise the proportion of patients completing the study and adhering to the low FODMAP-diet. In the control group we chose “no treatment”/“watchful waiting”, as the alternative. “Open-label placebo” has previously shown some efficacy on symptoms, which we aimed to minimise [33]. Still, the “no treatment/watchful waiting” in the control group was highly efficacious to decrease symptoms, suggesting a high placebo effect.

We followed the gold standard for testing food intolerance by eliminating and subsequently provoking in a blinded fashion as described above [14]. The amount of FODMAPs in the blinded provocation (25 g per day) was chosen to reflect the amount in a typical Danish diet, which is high compared to what has previously been reported from Australian (16 g per day) and American diets (12 g per day) [11,34]. The amount of FODMAPs in provocations fitted well with the amount diet patients had ingested before entering the study (20 g per day). In the study we provoked with a balance of all six types of FODMAPs to better mimic the real-life situation. Previous studies have provoked only with one or two FODMAPs, which rarely resembles a normal diet. The identical taste, look, scent and consistency of the provocation food supplements, efficiently prevented study personnel and patients from guessing the blinding status, which has previously been a problem in low-FODMAP diet studies [11]. Using food supplements instead of supplying all foods for the study allowed patients to live as normally as possible during participation in the study, to eliminate asocial eating behaviour to affect results.

In patients on an open-label low-FODMAP diet, abdominal pain scores decreased by 40% and bloating scores by 56%; this was similar to controls on watchful waiting. Provocations for two weeks with either placebo or FODMAPs reverted pain and bloating scores to baseline levels.

Patient recruitment problems resulted in a small sample size; 19 patients were included versus the calculated 45. Firstly, many patients chose not to complete the questionnaires, possibly resulting in selection bias, which could impair generalisability. Secondly, many patients with IBS according to questionnaire answers, actually experienced a flare or did not fulfil the ROME IV criteria when interviewed, resulting in a very low percentage of *IBS in IBD* in this study compared to previous data [35]. Among screened patients where both doctor and patient assessed those patients to be in remission, 8% had a flare documented at endoscopy. This calls for increased use of endoscopy in patients with UC experiencing abdominal pain or bloating but normal non-bloody stools.

Finally, as recruitment proved extremely difficult in this study, the primary focus changed to study feasibility and the final sample size reflected the largest feasible sample size at our two study hospitals and a crossover design was used to maximise power. Further, the primary outcome was changed to IBS-SSS as this is the gold standard scale for IBS symptoms. Recalculation of adequate sample size was not performed a priori, as the feasibility of simply completing the trial was the main goal. Additionally, within-person standard deviations were not available at the time. The initial power calculation for this RCT study was designed for a parallel trial with symptom diary as the primary outcome using data from a previous study in patients with IBD [15]. According to this calculation, a minimum difference in the symptom score on a VAS of 2.5, a standard deviation of 2.3 with 80% power and α = 0.05, 45 patients were needed for a parallel study. However, our study had a within-person SD of 48.4. Therefore, to detect a minimally relevant difference of 50% as recommended for IBS-SSS (i.e., 125.3 from baseline mean IBS-SSS of 247.5), a total of five patients were needed at 80% power and a two-sided alpha Level of 5%” [36]. The findings in this study regarding not significant differences in terms of blood and stool samples confirm other previous studies where no differences have been documented either.

A strength of the present study was that the gold standard was used for testing food intolerance with elimination followed by blinded provocations [14]. However, food provocations may result in a substantial nocebo response, which should be taken into account, and this may have influenced the results of our study [12]. In addition, the number of questionnaires should be reduced, as there is a large overlap between the results of the individual questionnaires.

The patient group was well defined and in deep remission, established by endoscopy, and histology before the study and calprotectin levels were monitored during the study, ensuring that the results did not reflect a new flare rather than the effect of the diet. Blood samples were analysed for C-reactive protein, white blood cell count, total iron, transferrin, ferritin, haemoglobin (whole blood), folate, cobalamin and red blood cell volume. Faeces was analysed for faecal calprotectin and bacterial DNA. There was no significant difference in any of the blood and microbiota samples except in Ferritin (*p* = 0.02). The paraclinical tests (calprotectin, blood and stool samples) were stable and documented continuing remission, thus not affecting the results. The existing literature on an association between low FODMAPs and microbiota changes is not unequivocal; this study showed no variations in the microbiota of study participants [11,13,37,38].

Surprisingly, the average pain score in the control group decreased by almost 50%, though not statistically significant, due to the low number and high variability of study participants. However, as these patients attended the same outpatient visits and completed the same questionnaires as the diet group, a placebo response was to be expected. Moreover, placebo responses up to 80% in short-lasting IBS trials have previously been observed [39]. This could not be explained by the controls adhering to the low FODMAP principles (monitored during the study), which constitutes a major challenge when planning future diet studies.

The open-label low-FODMAP diet resulted in a symptom decrease, which reflects either a dietary or a placebo effect. The placebo provocation also revealed a large nocebo effect. The nocebo effect resulting from a placebo provocation has previously been observed by Biesierski et al. and should be considered in future studies [12]. Provocation with FODMAPs elicited the same increase in symptoms as placebo provocation. The primary endpoint, which was the combined effect of elimination and subsequent provocation with FODMAPs, documented no change in IBS-SSS score, masking the pain decrease by the diet alone. In future studies, it will be necessary to measure an IBS-SSS score the day before starting the provocations.

## 5. Conclusions

This feasibility study provides insight into how to plan future diet studies. The results of the study document the possibility of performing a RCT following the gold standard for blindly testing food intolerance with the low-FODMAP diet. This feasibility study provides insight into how a future study such as this can be constructed.

The results of this blinded FODMAP RCT study in patients with UC in remission and comorbid symptoms of IBD suggested that placebo and nocebo responses explained the symptom dynamics when eliminating and subsequently provoking with FODMAPs.

## Figures and Tables

**Table 1:** Irritable Bowel Syndrome Severity Scoring System (IBS-SSS), Short Form 36 Health Survey (SF-36), Hospital Anxiety and Depression Scale (HADS), The Visceral Sensitivity Index (VSI), IBS specific Gastrointestinal Symptom Rating Scale (GSRS-IBS), Patient Health Questionnaire (PHQ 15), Irritable Bowel Syndrome Adequate Relief (IBS-AR).

Procedure	Screening	Visit 1 Baseline	Visit 2	Visit 3	Visit 4 Follow Up
Mayo Score	X				
Rome IV criteria	X			X	X
Dietician		X			
IBS-SSS		X	X	X	X
SF-36		X	X	X	X
HADS		X	X	X	X
GSRS		X	X	X	X
VSI		X	X	X	X
PHQ15		X	X	X	X
AR		X	X	X	X
FODMAP frequency (daily in the week before)		X	X	X	
Extra questions			X	X	X
Diet registration (3 days in the last week up to study start)		X	X	X	
Symptom diary	X-------------------------------------------------------------------------X				
Calprotectin	X	X	X	X	
Blood sample		X	X	X	
Body weight		X	X	X	X

**Table 2:** Baseline participant demographics.

Demographics	Control Group	Low FODMAP Diet
*n*	7	12
m/w	0/7	2/10
Age (median (IQR))	47 (42; 48)	38 (32; 50)
Caucasian	7/7	12/12
Weight (median (IQR)) kg	85.0 (54.2; 93.7)	72.4 (64.6; 87.2)
Debut year (median (IQR))	2010 (2009; 2012)	2012 (2005; 2016)
Family members with CU (%)	2 (29%)	3 (25%)
Smoker (%)	2 (29%)	1 (8.3%)
**Screening questionnaires**		
SCCAI (median (IQR))	3 (2; 5)	4 (3; 5)
Endo Mayo score (median (IQR)	1 (1; 1)	1 (1; 2)
*Missing (no endoscopy?)*	*2*	*3*
**Treatment**		
5-ASA treatment (%)	3 (43%)	5 (42%)
Azathioprine (%)	0 (0.0%)	1 (8.3%)
Biologics (%)	1 (14%)	1 (8.3%)
**Questionnaires at baseline, visits 2 and 3**		
IBS-SSS score (median (IQR))	273 (248; 280)	239 (208; 278)
Symptom score (average of 1 week) (median (IQR))	34.5 (28.5; 42.7)	15.8 (11.5; 25.8)
Bristol Stool Score (average of 1 week), median (IQR))	4.0 (3.1; 5.0)	5.0 (4.3; 5.2)
Stool frequency/day (average of 1 week)	2.1 (1.6; 2.3)	1.5 (1.1; 2.5)
GSRS score (median (IQR))	44 (36; 67)	45 (36; 50)
SF-36 score (median (IQR))	75.6 (50.0; 84.7)	76.1 (52.4; 82.1)
HADS score (median (IQR))	10 (5; 18)	9 (4; 14)
VSI score (median (IQR))	55 (29; 63)	54 (48; 65)
AR (% yes)	6 (85.7%)	3 (25.0%)
PHQ15 (median (IQR)	12 (9; 19)	11 (10; 16)
**Daily FODMAP intake**		
GOS	0.57 (0.54; 1.19)	0.73 (0.61; 0.78)
Fructans	4.87 (4.14; 5.57)	4.67 (3.84;4.80)
Fructose	1.23 (1.14; 2.14)	3.15 (1.37; 30.83)
Lactose	1.37 (0.70; 5.35)	10.19 (7.04; 11.64)
Sorbitol	0.36 (0.18; 2.04)	0.38 (0.14; 1.63)
Mannitol	0.03 (0.01; 0.08)	0.06 (0.06; 0.11)

Abbreviations: FODMAPS: Fermentable oligo-; di, and monosaccharides and polyols; n: Number; m: men; w: Women; IQR: Inter-quartile range; UC: Ulcerative colitis; SCCAI: Simple Clinical Colitis Activity Index; 5-ASA: 5-Aminosalicylates; IBS-SSS score: Irritable bowel symptom severity score; GSRS: IBS-specific Gastrointestinal Symptom Rating Scale; SF-36: Short Form 36 Health Survey; HADS: Hospital Anxiety and Depression Scale; VSI: The Visceral Sensitivity Index; AR: Irritable Bowel Syndrome Adequate Relief; PHQ15: Patient Health Questionnaire; GOS; GalactoOligoSaccharide.

**Table 3:** Results after intervention.

	Low FODMAP Diet			Control Group
Provocation	After FODMAP	After Placebo	*p*	After 4 Weeks
Guessed the blinding status	5 (56%)	3 (33%)	>0.99	-
Self-reported adherence to diet	9 (100%)	9 (100%)	-	-
Supplementary intake (median (IQR)) %	86% (74; 95)	93% (86; 100)	0.40	
Medication changes *	1 (11%)	0 (0.0%)	-	0 (0.0%)
**Secondary endpoints**, Median (IQR)				
Weight: kg, %	−2.0 (−3.1; −0.9)	−1.0 (−2.3; −0.5)	0.30	0.0 (−0.5; 0.2)
Change in Bristol Stool Scale score?	−0.3 (−1.0; 0.3)	−0.1 (−0.5; 0.0)	0.30	0.3 (−0.2; 0.5)
Change in stool frequency/day	0.0 (−0.9; 0.4)	−0.2 (−0.6; 0.0)	0.20	0.3 (0.2; 0.5)
Change in GSRS score	−6 (−17; −3)	−7 (−11; −1)	0.19	−4 (−7; −0)
Change in SF-36 score	3.2 (1.1; 7.5)	2.2 (−3.5; 4.3)	0.24	−5.7 (−10.3; 0.3)
Change in HADS score	−2 (−6; −2)	0 (−4; 0)	0.03	0 (−2; 2)
Change in VSI score	6 (3; 11)	−1 (−6; 7)	0.29	−3 (−4; 3)
Change in AR: No to Yes, % Yes to No, %	3 (33%) 0 (0.0%)	3 (33%) 0 (0.0%)	1.00	0 (0.0) 3 (42.9)
Change in PHQ15	−4 (−5; −2)	−4 (−4; −1)	0.67	0 (−4; 2)
**Change in FODMAPS in diet registration**, %				
Index for all FODMAPs	−49% (−71; −37)	−74% (−78; −36)	0.81	33% (2%; 49%)
Fructose	−38% (−49; −30)	−58% (−67.5; −20)	1.00	−13% (−29%; 37%)
Lactose	−96% (−99; −80)	−99% (−99; −94)	1.00	166% (141%; 287%)
Sorbitol	−58% (−93; 147)	−91% (−100; 44)	0.06	−34% (−54%; 81%)
Mannitol	−22% (−67; −17)	−28% (−50; 9.1)	1.00	200% (−10%; 325%)
Fructans	−32% (−47; −29)	−31% (−50; −31)	0.81	0% (−26%; 23%)
GOS	−43% (−70; −23)	−40% (−52; −20)	0.63	−3% (−19%; 7%)
**Change in FODMAPS in frequency registration**, %				
Fructose	−30% (−73; 39)	−93% (−97; −90)	0.06	−15% (−52%; 166%)
Lactose	−100% (−100; −66)	−100% (−100; −57)	0.58	−7% (−28%; 96%)
Sorbitol	−83% (−92; −77)	−97% (−100; −80)	0.69	20% (−25%; 196%)
Mannitol	−98% (−100; −37)	−100% (−100; −86)	0.42	−35% (−66%; 24%)
Fructans	−90% (−96; −56)	−89% (−93; −76)	0.56	1% (−10%; 39%)
GOS	−83% (−98; −37)	−54% (−84; −13)	0.69	−1% (−33%; 5%)

Abbreviations: FODMAPS: Fermentable oligo-, di, and monosaccharides and polyols; n: Number; m: men; w: Women; IQR: Inter-quartile range; UC: Ulcerative colitis; SCCAI: Simple Clinical Colitis Activity Index; 5-ASA: 5-Aminosalicylates; IBS-SSS score: Irritable bowel symptom severity score; GSRS: IBS-specific Gastrointestinal Symptom Rating Scale; SF-36: Short Form 36 Health Survey; HADS: Hospital Anxiety and Depression Scale; VSI: The Visceral Sensitivity Index; AR: Irritable Bowel Syndrome Adequate Relief; PHQ15: Patient Health Questionnaire; GOS; GalactoOligoSaccharide. * started 5-ASA treatment during FODMAP provocation.

## Data Availability

The data presented in this study are available on request from the corresponding author.

## Supplementary Materials

The following supporting information can be downloaded at: https://www.mdpi.com/article/10.3390/nu14061296/s1, Figure S1: Gut microbiota of the different patient groups.
