# Supplementary figures and images for: Efficacy of FODMAP Elimination and Subsequent Blinded Placebo-Controlled Provocations in a Randomised Controlled Study in Patients with Ulcerative Colitis in Remission and Symptoms of Irritable Bowel Syndrome: A Feasibility Study
Source: Nutrients. 2022 Mar 18;14(6):1296. doi: 10.3390/nu14061296 (PMC8955641; doi:10.3390/nu14061296)

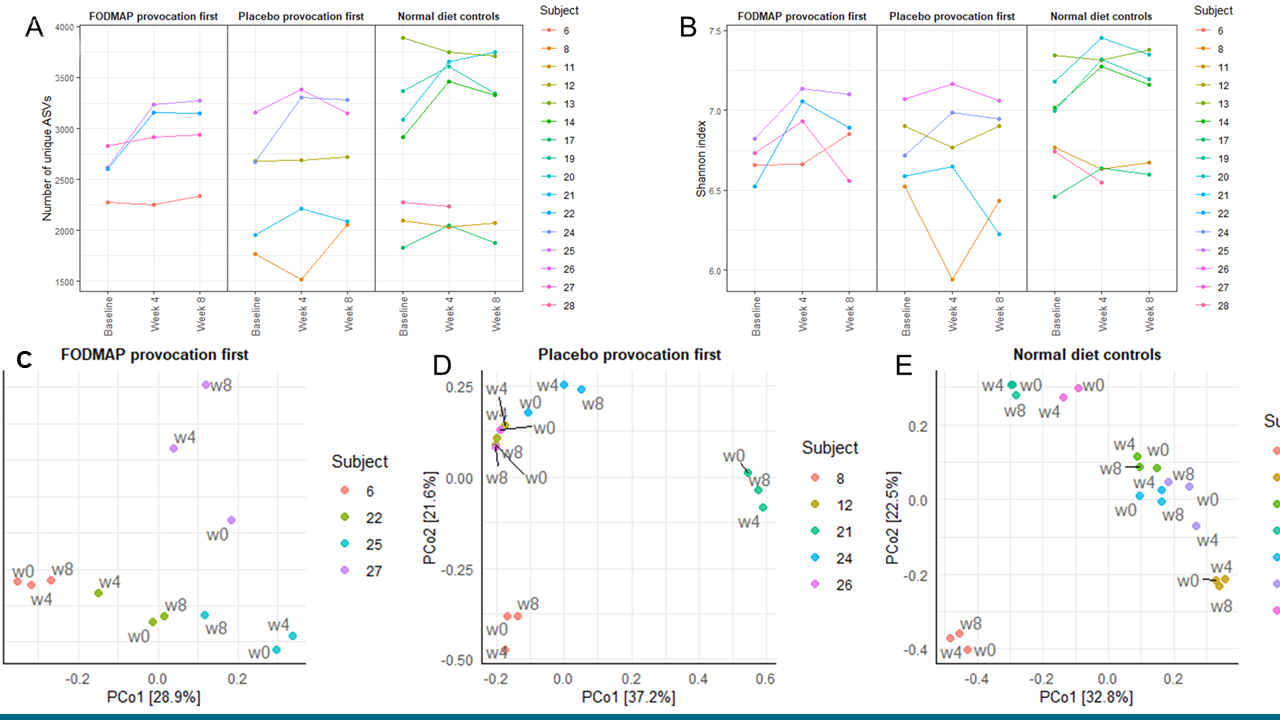

Supplement: Supplementary file 1 [file nutrients-14-01296-s001.zip › nutrients-1599910-supplementary.TIF]
